# Medical College of the State of South Carolina

**Published:** 1845-08

**Authors:** 


### MEDICAL COLLEGE OF THE STATE OF SOUTH CAROLINA.

We have received the "Catalogue of the Trustees, Faculty and Students," published the present year, from which it appears that the class of last Session numbered one hundred and ninety-six, seventy-four of whom received the Degree of Doctor of Medicine, at the last annual commencement. The distinguished gentlemen who have constituted the faculty for several years past, continue to occupy their chairs as heretofore.

